# Efficacy and Safety of Enteral Human Recombinant Insulin to Reduce the Time to Full Enteral Feeding in Preterm Infants: A Meta-Analytical Study

**DOI:** 10.3390/pediatric15020033

**Published:** 2023-06-07

**Authors:** Faiza Qudsia, Muhammad Adil, Maha Kamran, Muhammad Azam, Huzaifa Ahmad Cheema, Abia Shahid, Ivan Cherrez-Ojeda

**Affiliations:** 1 Department of Medicine, King Edward Medical University, Lahore 54000, Pakistan; f.qudsia48@gmail.com (F.Q.);; 2 Department of Medicine, Allama Iqbal Medical College, Lahore 54550, Pakistan; 3 Department of Paediatrics, King Edward Medical University, Lahore 54000, Pakistan; 4 Respiralab Research Center, Guayaquil 090512, Ecuador; 5 School of Medicine, Universidad Espíritu Santo, Guayaquil 092301, Ecuador

**Keywords:** enteral insulin, preterm infants, enteral feeding, meta-analysis

## Abstract

Recombinant human insulin plays an important role in the gut maturation of preterm infants. This meta-analysis was carried out to assess the efficacy and safety of enteral recombinant human insulin in decreasing the time to full enteral feeding in preterm infants. The pooling of data from four clinical trials yielded a significant decrease in the time to full enteral feeding in preterm infants under both low (Mean difference [MD] −3.43 days; 95% CI: −6.18 to −0.69 days; I2 = 48%) and high doses of insulin (MD −7.10 days; 95% CI: −10.02 to −4.18 days; I2 = 0%). These findings require confirmation by further large trials that evaluate the efficacy and safety of enteral insulin, especially at supraphysiological doses.

## 1. Introduction

A preterm birth is categorized as the birth of a baby before 37 weeks of gestation [1]. Around 15 million children are born prematurely every year, representing a worldwide rate of 11% [2,3]. Preterm infants often have low birth weight and feeding problems associated with immaturity of the gastrointestinal system. The full development of the gastrointestinal tract, including motor development and lactase activity, typically occurs in the third trimester of pregnancy [4]. As a result, preterm infants may require enteral feeding via naso- or orogastric tubes to support their nutritional needs; however, gut immaturity makes enteral feedings difficult to tolerate. Feeding intolerance may include clinical manifestations such as vomiting, abdominal distension, and gastric residuals [5]. The consequence of feeding intolerance is increased dependency on total parenteral nutrition (TPN) and an increased risk of associated complications such as intestinal-associated liver disease and late-onset sepsis [6,7,8]. Therefore, interventions that improve feeding intolerance and enhance a quick transition to full enteral feeding in preterm infants are crucial.

Studies on animal models suggest that maternal milk contains factors that stimulate the development of the gastrointestinal tract [9]. These intestinal-enhancing effects of maternal milk are attributed to a multitude of non-nutritive, biologically active factors, including the peptide hormone insulin [10]. During the early puerperal period, insulin levels peak and then decline to a basal level within 3 days of postpartum [11]. Unfortunately, insulin is not yet available in formula powders.

Some clinical trials have been conducted on this topic, but they have not been systematically evaluated. In this study, we aim to evaluate the efficacy and safety of enteral human recombinant insulin in reducing the time to full enteral feeding in preterm infants. Our meta-analysis will help to determine if this intervention can prevent feeding intolerance and associated complications in this vulnerable population.

## 2. Methods

This meta-analysis was conducted according to the guidelines of the Preferred Reporting Items for Systematic Review (PRISMA) [12]. The PRISMA guidelines checklist has been submitted as Supplementary Materials for this journal article. We thoroughly searched all relevant articles on PubMed and Embase from inception until 20 December 2022 using the following search terms: (“Insulin”) AND (“preterm” OR “LBW” OR “Low birth weight” OR “birth weight” OR “infants”) AND (“Feeding intolerance” OR “Maturation” OR “full enteral feed” OR “enteral feed”). We applied no language restrictions.

We initially screened articles based on titles and abstracts and then screened them by reading the full text of the research articles. Two separate authors (FQ and MA) conducted the screening, and any conflicts and discrepancies were resolved through discussion with the third author (MA). We included controlled clinical trials that enrolled preterm infants and compared the efficacy of recombinant human insulin concentration with placebo or no treatment. Our outcomes of interest were the reduction in the time to full enteral feeding and the incidence of serious adverse events.

We pooled the data using RevMan 5.4 software under a random-effects model using mean differences (MDs) and risk ratios (RRs) with their 95% confidence intervals (CIs). Heterogeneity was assessed by the I^2^ statistic. We evaluated the efficacy of enteral insulin in two doses: (a) the physiological dose (400 µIU/mL milk) present in human colostrum (low dose) and (b) a supraphysiological dose (2000 µIU/mL milk) (high dose) [11].

## 3. Results

**Figure 1:**
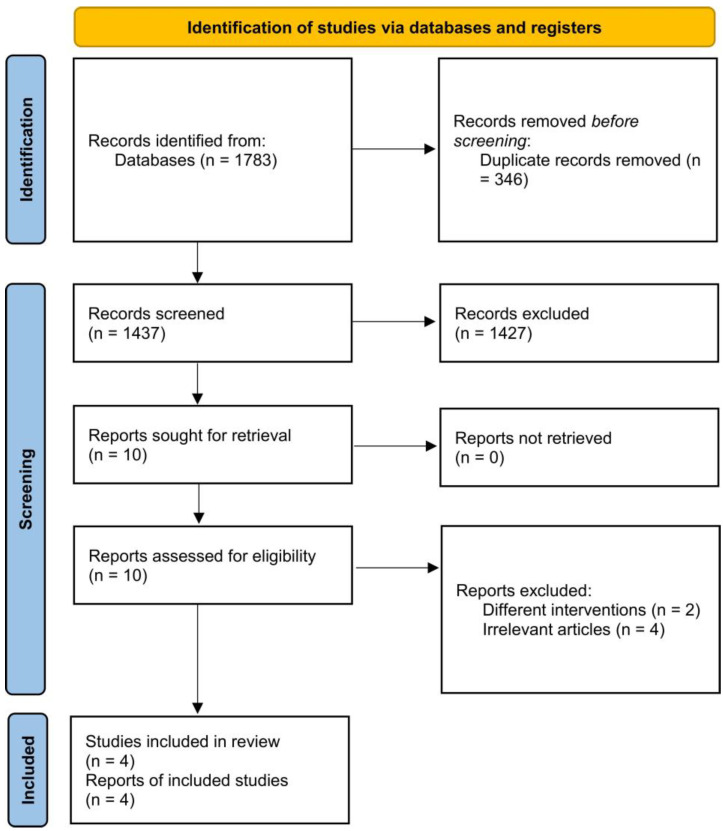
PRISMA 2020 flowchart.

The screening process identified four trials meeting the inclusion criteria that contributed five comparisons to the meta-analysis with a total of 388 participants (204 receiving enteral recombinant insulin, 184 receiving placebo) [13,14,15,16]. The detailed screening process is depicted in Figure 1. Three studies were randomized controlled trials (RCTs) [13,14,16] while the remaining trial was a pilot study that used a matched historical cohort as the comparator [15]. Two studies used a low dose of insulin [13,14], one study evaluated a supraphysiological dose of insulin [15], and the remaining RCT assessed both low and high doses [16]. The characteristics of each study are summarized in Table 1.

**Figure 2:**
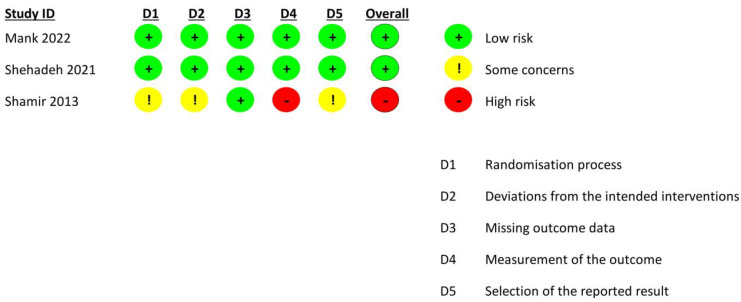
Quality assessment of included trials [13,14,16].

The quality assessment of RCTs was carried out using the revised Cochrane Risk of Bias (RoB 2.0) tool [17]. One trial was at a high risk of bias while the other two were judged to have a low risk of bias (Figure 2). The pilot study was rated as fair using the National Institute of Health’s (NIH) quality assessment tool (Table 2) [18].

**Figure 3:**
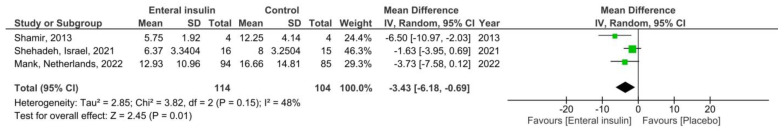
Effect of low-dose insulin on time to full enteral feeding [13,14,16].

**Figure 4:**
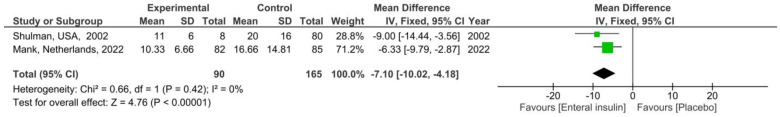
Effect of high-dose insulin on time to full enteral feeding [13,16].

**Figure 5:**
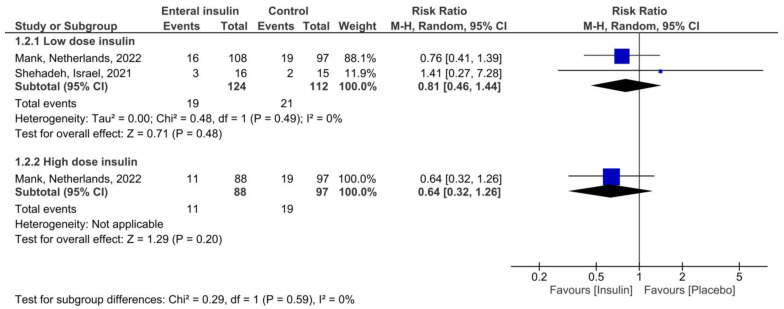
Incidence of adverse events with enteral insulin [13,16].

The results of our meta-analysis show that the use of enteral recombinant human insulin significantly decreased the time to full enteral feeding in preterm infants at both low (MD −3.43 days; 95% CI: −6.18 to −0.69 days; I^2^ = 48%; Figure 3) and high doses (MD −7.10 days; 95% CI: −10.02 to −4.18 days; I^2^ = 0%; Figure 4). Upon sensitivity analysis after excluding the study by Shamir et al. in the low-dose group, the heterogeneity decreased to 0% potentially due to the study’s small sample size. Because only one study reported serious adverse events in the high-dose group, indicating that high-dose insulin was safe (RR 0.64; 95% CI: 0.32 to 1.26), adverse events were meta-analyzed in the low-dose insulin group only. Low-dose insulin did not increase the number of serious adverse events (RR 0.81; 95% CI: 0.46 to 1.44; I² = 0%; Figure 5).

## 4. Discussion

To the best of our knowledge, this is the first meta-analysis that focuses on the use of enteral recombinant insulin in preterm infants. Our results indicate that the use of enteral insulin in preterm infants promotes gut maturation and increases enteral feeding intake. Our findings demonstrate that both low- and high-dose insulin significantly reduce the time to achieve full enteral feeding. There was no significant difference in the incidence of serious adverse events between the insulin and control groups. The occurrence of adverse events was largely attributed to the preterm birth of infants.

Managing preterm infants is challenging because it involves preventing sepsis and necrotizing enterocolitis, which are common in these infants. Preterm infants may need to receive their nutrition through nasogastric or orogastric tubes due to an inability to feed orally resulting from neurological immaturity [19]. However, feeding intolerance is a common condition in this population. The development of the gastrointestinal tract, including the ability to move and digest lactose, usually happens entirely during the third trimester of pregnancy. Gut immaturity and decreased lactase activity contribute to feeding intolerance [5]. To address this, factors that promote lactase activity can be used. Human milk contains various bioactive factors, hormones, and nutrients that protect infants. However, preterm infants who cannot tolerate full enteral feeding should have their nutritional requirements covered parenterally. This can increase the length of hospitalization, and subsequently, the risk of hospital-acquired infections, especially in developing countries. Hence, any intervention that decreases the time to full enteral feeding can directly reduce the duration of hospital stay and decrease the complications associated with parenteral feeding.

Some limitations of our meta-analysis need to be considered. Most of our included trials were small with only one study involving more than 80 participants in each group. Furthermore, one of the pooled studies was a pilot study that used a historical cohort as the comparator. There are limited data about the safety of enteral insulin, especially with high doses of insulin.

In summary, our study supports the use of insulin in preterm infants as it reduces the time to full enteral feeding. This provides a new direction for the management of preterm neonates, contributing to a decreased duration of hospital stay and the prevention of short-term and long-term consequences of hospitalization. However, further larger studies are required to evaluate the efficacy and safety of insulin, especially at supraphysiological doses. Furthermore, the effect of this intervention on other clinical outcomes reflecting reduced morbidity should be evaluated. Additionally, the efficacy of enteral recombinant human insulin in extremely preterm infants (born at gestational ages of less than 28 weeks) should be evaluated.

## Figures and Tables

**Table 1:** Characteristics of the included studies.

First Author, Year, Country	Main Inclusion Criteria	Number of Randomized Neonates (n)	Initiation and Duration of the Study Intervention	Mean Gestational Age (SD)/Median (IQR) Weeks	Mean Birth Weight (SD), Median (IQR) g	Primary Outcome(s)	Secondary Outcome(s)	Other Reported Outcome(s)
Shulman RJ, 2002, USA [15]	26–30 weeks of gestation, appropriate size for gestational age; postnatal age < 96 h	Insulin Group = 8, Control Group = 80	Insulin administration was initiated on the 4th day of life and continued until the 28th day of life. Intestinal lactase activity in both groups of neonates was determined at 28 days of age.	Insulin Group 27.8 (2.5), Control Group 27.8 (1.2)	Insulin Group 973 (310), Control Group 1042 (172)	Intestinal lactase activity Mean number of neonates with gastric residuals > 2 mL/kg	-	-
Shamir R, 2013, Israel [14]	Preterm Infants	8 neonates	Day 1–28 after birth	27.8 (2.5)	800 and above	Weight gain and time to achieve FEF	-	-
Shehadeh N, 2021, Israel [13]	Preterm Infants at 26–33 weeks of gestation, birth wt. ≥ 750 g, postnatal age ≤ 7 days	33 preterm infants	Within 24 h of enrollment for 28 days or until the time of discharge, whichever was sooner	Placebo 30.6 ± 2.1, Insulin 30.9 ± 1.5	Placebo 1446.8 ± 364.8, Insulin 1470.7 ± 299.7	Time required to achieve FEF	Severe adverse effects	Weight gain in the first 28 days after birth
Mank E, 2022, Netherlands [16]	26–32 weeks of gestation, weight of 500 g or more, able to tolerate enteral feeding	303 neonates	Within 5 days post-partum (up to 120 h). In the neonates exclusively fed with mother’s milk, treatment was not initiated until 72 h post-partum. The standard duration of the intervention was 28 days	Low-dose insulin 29.1 (28.1–30.4), High-dose insulin 29.0 (27.7–30.5), Placebo 28.8 (27.6–30.4)	Low-dose group 1200 (976–1425), High-dose group 1250 (1020–1445), Placebo group 1208 (1021–1430)	Time to achieve full enteral feeding (FEF) defined as an enteral intake of 150 mL/kg per day or more for 3 consecutive days.	The proportion of neonates who achieved FEF in the first 6, 8, and 10 days of intervention	Assessment of severe adverse Effects after the administration of different doses of enteral insulin.

SD, Standard Deviation; IQR, Interquartile Range.

**Table 2:** Quality assessment of the pilot study.

Criteria	Shulman RJ 2002, USA [15]
Was the research question or objective in this paper clearly stated?	✔
Was the study population clearly specified and defined?	✔
Was the participation rate of eligible persons at least 50%?	✔
Were all the subjects selected or recruited from the same or similar populations?	✔
Was a sample size justification, power description, or variance and effect estimates provided?	✔
For the analyses in this paper, were the exposure(s) of interest measured prior to the outcome(s) being measured?	✔
Was the timeframe sufficient so that one could reasonably expect to see an association between exposure and outcome if it existed?	✔
For exposures that can vary in amount or level, did the study examine different levels of the exposure?	NA
Were the exposure measures (independent variables) clearly defined, valid, reliable, and implemented consistently across all study participants?	✔
Was the exposure(s) assessed more than once over time?	NA
Were the outcome measures (dependent variables) clearly defined, valid, reliable, and implemented consistently across all study participants?	✔
Were the outcome assessors blinded to the exposure status of participants?	CD
Was loss to follow-up after baseline 20% or less?	✔
Were key potential confounding variables measured and adjusted statistically for their impact on the relationship between exposure(s) and outcome(s)?	No
Summary Quality	Fair

NA, not applicable; CD, cannot decide.

## Data Availability

The data that support the findings of this study are available from the corresponding author upon reasonable request.

## Supplementary Materials

The following supporting information can be downloaded at: https://www.mdpi.com/article/10.3390/pediatric15020033/s1.
